# The systemic immune response due to cholesterol crystal embolization syndrome: a case report

**DOI:** 10.1186/s12882-022-02939-9

**Published:** 2022-09-19

**Authors:** Tetsu Sakamoto, Takafumi Yamakawa, Keita Hirano, Arisa Kobayashi, Mio Kasai, Kiyoshi Koizumi, Takashi Yokoo, Satoru Komatsumoto, Toshimitsu Murohisa, Taro Shimizu

**Affiliations:** 1 Department of Internal Medicine, Japanese Red Cross Ashikaga Hospital, Yobe-cho 284-1, Ashikaga, Tochigi, 326-0843 Japan; 2 Department of Diagnostic and Generalist Medicine, Dokkyo Medical University, Mibu-cho, Simotsuga-gun, Tochigi, Japan; 3 Division of Nephrology and Hypertension, Department of Internal Medicine, The Jikei University School of Medicine, Minato-ku, Tokyo, Japan; 4 Department of Cardiovascular Surgery, Japanese Red Cross Ashikaga Hospital, Yobe-cho 284-1, Ashikaga, Tochigi, 326-0843 Japan

**Keywords:** Cholesterol embolization, Vancomycin, Acute tubulointerstitial nephritis, Acute kidney injury

## Abstract

**Background:**

Cholesterol crystal embolization syndrome (CES) occurs when an atherosclerotic plaque causes small-vessel embolization, resulting in multi-organ damage. Although CES is pathologically characterized by an infiltration of eosinophils, the implication of the systemic inflammatory response represented by hypereosinophilia is unclear in clinical practice. Herein we present the case of a patient diagnosed with CES who developed multiple allergic organ injuries, including daptomycin-related dermatitis and later vancomycin-induced acute tubulointerstitial nephritis, which was successfully treated by the withdrawal of each medicine with or without corticosteroid therapy, one by one.

**Case presentation:**

A 76-year-old Japanese man diagnosed with thoracic aneurysm rupture underwent total arch replacement through the open stent graft technique. Postoperatively, he developed methicillin-resistant *Staphylococcus epidermidis* bacteremia, which was treated with daptomycin. Subsequently, he presented with palpable purpura on both dorsal feet, erythema around his body, and hypereosinophilia. Daptomycin was replaced with vancomycin due to suspicion of drug-induced erythema. The erythema gradually faded. On nine days after vancomycin therapy, the systemic erythema rapidly reappeared followed by acute renal failure. The renal function decline prompted hemodialysis. A skin biopsy revealed cholesterol embolization, whereas a kidney biopsy revealed acute tubulointerstitial nephritis. After vancomycin discontinuation and initiation of systemic corticosteroid treatment, his kidney function was restored to the baseline level.

**Conclusions:**

The present case highlights cholesterol embolization can cause allergic complications in addition to direct organ damage.

## Background

Cholesterol crystal embolization syndrome (CES) is a disease characterized by small-vessel embolization due to the collapse of an atherosclerotic plaque in larger vessels induced by vascular manipulation. The cascade of cholesterol crystal embolization in small arteries can damage the kidneys, gastrointestinal tract, central nervous system, retina, and other organs [1]. The embolization process induces a localized endothelial inflammatory response in the affected small arteries. Notably, the early phase of CES is characterized by pathologically-identified eosinophil and polymorphonuclear infiltrate. However, the implication of the systemic inflammatory response represented by hypereosinophilia, which has been observed in 80% of patients with CES, is unclear in clinical practice [1]. Herein we present the case of an elderly patient with daptomycin-related dermatitis and acute kidney injury (AKI) due to vancomycin-induced acute tubulointerstitial nephritis and CES.

## Case presentation

**Fig. 1:**
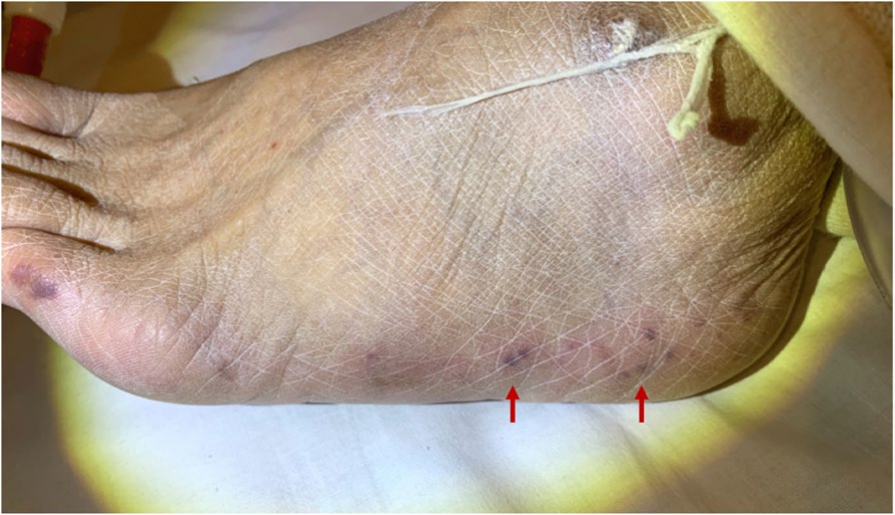
Palpable purpura on the left dorsal foot (arrow)

**Fig. 2:**
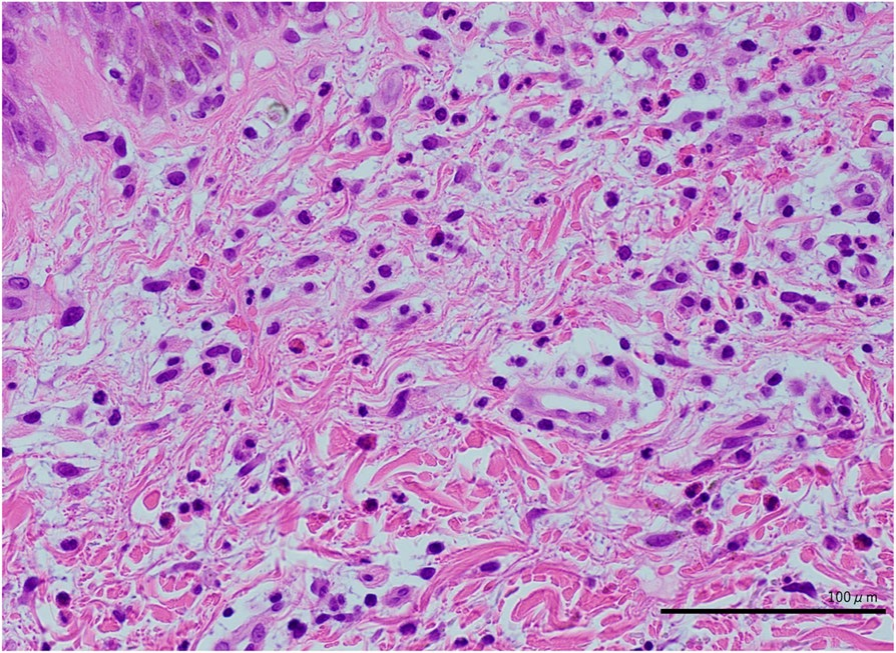
Histopathology of the erythema, which exhibited inflammatory cell infiltration into the dermis (Hematoxylin–eosin, 400 ×)

**Fig. 3:**
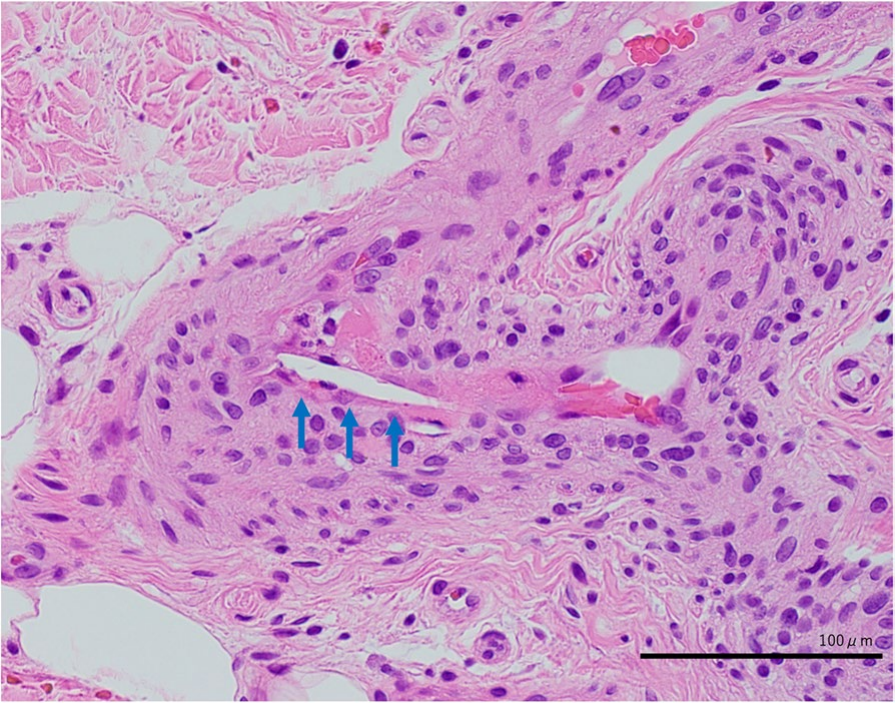
Histopathology of the palpable purpura, which exhibited a cleft in a small artery (arrow; Hematoxylin–eosin, 400 ×)

**Fig. 4:**
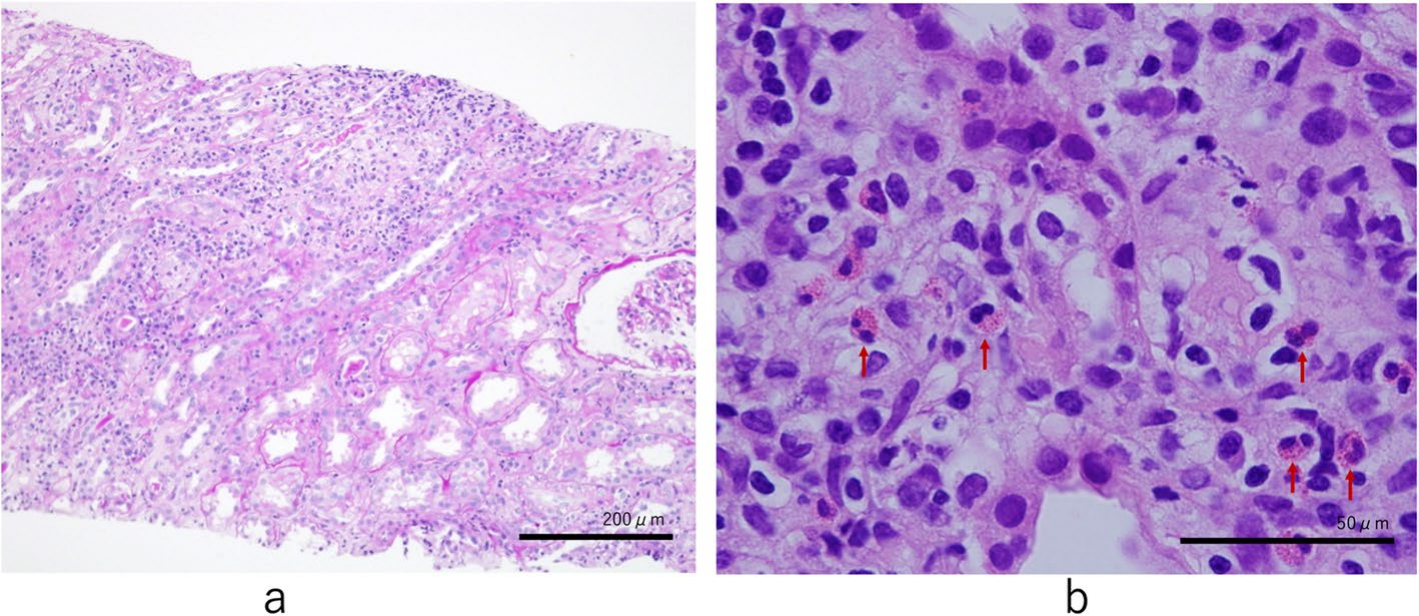
Histopathology of the kidney with tubulointerstitial nephritis with eosinophil infiltration (arrow) (**a**) Periodic acid–Schiff, 100 × and (**b**) Hematoxylin–eosin, 1000 × , respectively

**Fig. 5:**
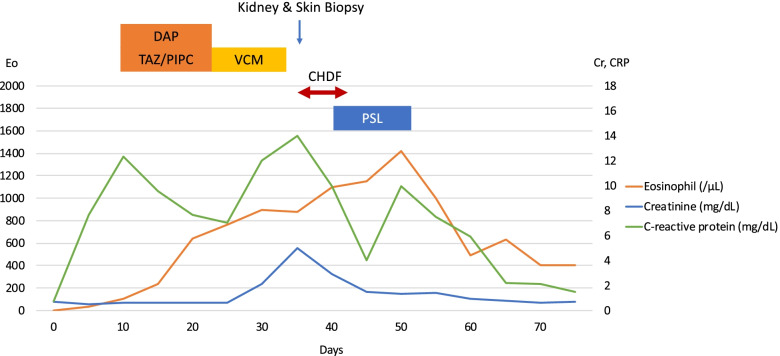
Clinical course including blood biochemistry data. Eo, eosinophil; Cr, creatinine; CRP, c-reactive protein; PSL, prednisolone; VCM, vancomycin; DAP, daptomycin; TAZ/PIPC, tazobactam/piperacillin; CHDF, continuous hemodiafiltration

A 76-year-old man was admitted for an acute thoracic aneurysm rupture and underwent an emergent total arch replacement through the open stent graft technique. Past medical history included hypertension and alcoholic hepatitis. There was no history of asthma or atopic dermatitis and no drug allergy episodes. On postoperative day 7, his sputum increased, and wet and pan-inspiratory crackles presented bilaterally on auscultation of the lungs. Laboratory examination showed elevated C-reactive protein levels, and chest X-ray revealed ground-glass opacity over both lungs. The patient was diagnosed with ventilator-associated pneumonia and treated with tazobactam/piperacillin at 4.5 g three times daily. Two sets of blood cultures obtained on day 7 grew methicillin-resistant coagulase-negative staphylococci. Considering the nephrotoxicity of the combination of vancomycin and tazobactam/piperacillin, the patient was started on daptomycin at 300 mg daily. There was no vegetation on echocardiography. On day 21, the patient presented with disseminated body erythema and palpable purpura on both of his dorsal feet as well as unexpectedly increased eosinophils up to 600/μL from 100/μL. Daptomycin was replaced with vancomycin at 2 g daily with regular monitoring of the serum trough concentration, suspicion of drug-induced erythema. The purpura persisted, but the erythema gradually faded. On postoperative day 30, the erythema rapidly reappeared on his trunk, upper extremities, and bilateral thighs. There was also palpable purpura on the lateral border of the soles (Fig. 1). The serum creatinine level increased up to 5.16 mg/dL, and the serum trough concentration of vancomycin increased to 59 mg/L. Serum eosinophils were as high as 1100/μL. Urinalysis revealed 4.5 g/gCr of proteinuria and isomorphic hematuria. Urine β_2_-microglobulin was 15,700 μg/L, and urine N-acetyl glucosaminidase was 21.8 U/L. Tests for serum antinuclear and antineutrophil cytoplasmic antibodies were negative. An abdominal computed tomography without contrast showed a distended kidney with urinary tract obstruction. Echocardiography revealed no evidence of vegetation. Repeated blood and urine cultures were negative. Skin biopsies of the erythema and palpable purpura confirmed the infiltration of eosinophils and other inflammatory cells into the dermis (Fig. 2). The biopsy of the purpura also showed clefts in the small vessels, with the presence of inflammatory cell infiltrates around these clefts, consisting mainly of polymorphonuclear cells (Fig. 3). A kidney biopsy revealed acute tubulointerstitial nephritis with eosinophil infiltration (Fig. 4a and b). Indirect fluorescent antibody technique findings were inconclusive. In summary, a skin biopsy revealed cholesterol embolization, whereas a kidney biopsy revealed acute tubulointerstitial nephritis. An Olympus BX51 was used for optical microscopy, and DPController was used as the imaging software. The measured resolution was 4080 × 3072. Vancomycin was discontinued and the patient underwent emergent hemodialysis for AKI. His renal function did not worsen further; however, it remained impaired. Prednisolone 20 mg daily was given for 11 days beginning on day 39, on which treatment the erythema gradually resolved. Over 30 days, the patient’s serum creatinine level decreased from 5 mg/dL to 0.7 mg/dL at baseline without hemodialysis. Serum eosinophils were improved, and no new skin rash developed (Fig. 5). The patient was eventually discharged from the hospital.

## Discussion and conclusions

The present case report demonstrated a pathologically evidenced CES accompanied by daptomycin-related dermatitis, vancomycin-induced acute tubulointerstitial nephritis, and AKI in the context of total arch replacement through the open stent graft technique for the thoracic aneurysm rupture.

The intriguing teaching point of this case is that CES potentially caused several allergic events. Cholesterol crystals are known to cause inflammatory reactions around arterioles besides vascular occlusion [1]. The previous study showed cholesterol crystals induce inflammation via nucleotide-binding oligomerization domain-like receptor family, pyrin domain-containing 3 (NLRP3) inflammasome, resulting in caspase-1 activation and interleukin (IL)-1β secretion [2]. This reaction then leads to an increase in eosinophils [3, 4]. Previous reports have indicated that eosinophilia occurs in 80% of patients, which might be a clue to the diagnosis of CES [1, 5]. The commonly found eosinophilia in CES could progress to further allergic organ injury. Meanwhile, the allergic organ events in CES might have been underdiagnosed. For instance, there is a case report from France of CES with erythroderma after allopurinol initiation, in which the authors practically described that, in the beginning, they could not diagnose CES but were misled to a diagnosis of adverse drug reaction with eosinophilia and systemic symptoms [6]. The present case may have one more implication to the clinical management in CES, because, once eosinophilia in CES had been overt, not singe but multiple medications would breed to allergic complications, including daptomycin-associated dermatitis and vancomycin-induced acute tubulointerstitial nephritis. In addition, before those allergic complications in CES, there has been a suggestion of a baseline association between allergic diseases and atherosclerosis [7]. As far as we could find, there are no reports of multiple allergic complications before or after the onset of CES. But taken together, it would be speculated that this inflammation led to a breeding ground for the subsequent allergic complications.

We assumed that the major cause of AKI was diffuse tubulointerstitial nephritis, triggered by the administration of vancomycin, not daptomycin, after the onset of systemic allergy in CES. AKI only became apparent nine days after vancomycin administration, the concentration of which reached a level previously reported to be a high risk for vancomycin-related nephropathy. Vancomycin and daptomycin have different effects on renal tubular cells. In tobramycin kidney injury studies, daptomycin reduced renal damage, whereas vancomycin did not exhibit renal protective effects [8, 9]. Previous clinical studies showed higher rates of AKI in patients treated with vancomycin than that in those treated with daptomycin (odds ratio of 4.4 in one study) [10, 11]. In addition, the pathology of vancomycin-induced nephropathy has been reported to exhibit acute interstitial nephritis and acute tubular necrosis [12]. Considering the above information, vancomycin was considered more probable than daptomycin as the suspect drug for the AKI in the present patient. Drug-induced allergic reactions developed cutaneous and renal injury during a typical CES in this case. CES has the possibility of causing allergic complications besides direct organ damage. Although further research is needed, we believe this case is an important stepping stone.

## Data Availability

The datasets used in this case are available from the corresponding author on reasonable request.

## Abbreviations

CES: Cholesterol crystal embolization syndrome
AKI: Acute kidney injury
NLRP3: Nucleotide-binding oligomerization domain-like receptor family, pyrin domain-containing 3
